# ATP–Magnesium Coordination: Protein Structure-Based Force Field Evaluation and Corrections

**DOI:** 10.1021/acs.jctc.0c01205

**Published:** 2021-02-22

**Authors:** Floris P. Buelens, Hadas Leonov, Bert L. de Groot, Helmut Grubmüller

**Affiliations:** † Department of Theoretical and Computational Biophysics, Max Planck Institute for Biophysical Chemistry, Göttingen 37077, Germany; ‡ Computational Biomolecular Dynamics Group, Max Planck Institute for Biophysical Chemistry, Göttingen 37077, Germany

## Abstract

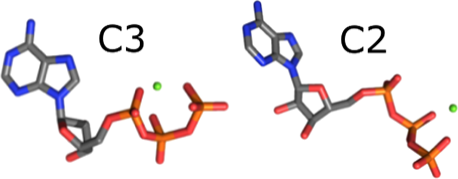

In the numerous molecular recognition and catalytic processes across biochemistry involving adenosine triphosphate (ATP), the common bioactive form is its magnesium chelate, ATP·Mg^2+^. In aqueous solution, two chelation geometries predominate, distinguished by bidentate and tridentate Mg^2+^–phosphate coordination. These are approximately isoenergetic but separated by a high energy barrier. Force field-based atomistic simulation studies of this complex require an accurate representation of its structure and energetics. Here we focused on the energetics of ATP·Mg^2+^ coordination. Applying an enhanced sampling scheme to circumvent prohibitively slow sampling of transitions between coordination modes, we observed striking contradictions between Amber and CHARMM force field descriptions, most prominently in opposing predictions of the favored coordination mode. Through further configurational free energy calculations, conducted against a diverse set of ATP·Mg^2+^–protein complex structures to supplement otherwise limited experimental data, we quantified systematic biases for each force field. The force field calculations were strongly predictive of experimentally observed coordination modes, enabling additive corrections to the coordination free energy that deliver close agreement with experiment. We reassessed the applicability of the thus corrected force field descriptions of ATP·Mg^2+^ for biomolecular simulation and observed that, while the CHARMM parameters display an erroneous preference for overextended triphosphate configurations that will affect many common biomolecular simulation applications involving ATP, the force field energy landscapes broadly agree with experimental measurements of solution geometry and the distribution of ATP·Mg^2+^ structures found in the Protein Data Bank. Our force field evaluation and correction approach, based on maximizing consistency with the large and heterogeneous collection of structural information encoded in the PDB, should be broadly applicable to many other systems.

## Introduction

Hydrolysis of adenosine triphosphate (ATP) serves as a source of chemical energy across all domains of life, driving essentially all energy-consuming cellular processes. Both unbound in cellular surroundings and as enzyme substrates, nucleotide triphosphates occur predominantly with divalent cations, most commonly Mg^2+^,^1,2^ coordinated with the negatively charged triphosphate moiety. Given its ubiquity, an accurate steric and energetic description of the ATP·Mg^2+^ complex, including its cation–phosphate interactions, is key to theoretical studies and atomistic simulations of many ATP-consuming processes.

**Figure 1:**
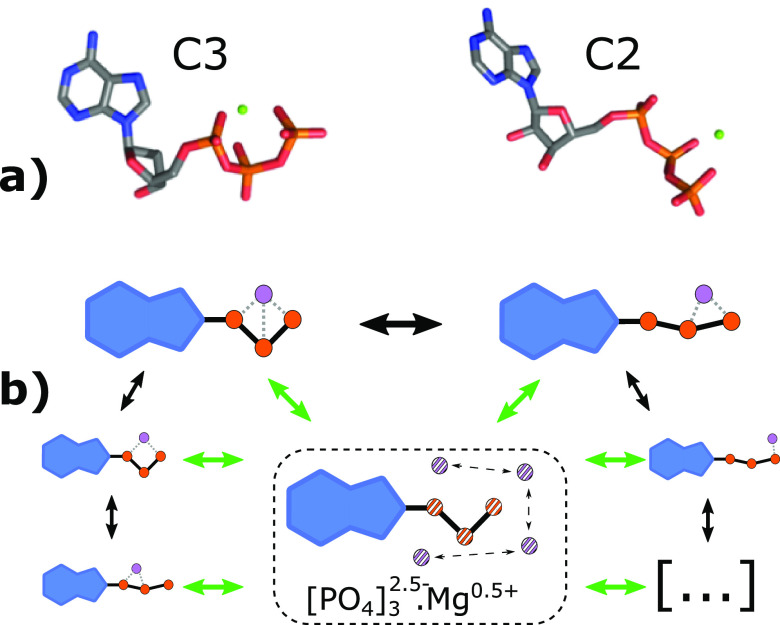
**a)** ATP·Mg^2+^ in tridentate (triple Mg^2+^–phosphate coordination, C3) and bidentate (double, C2) configurations. **b)** Free energy calculation scheme. Conversion between coordination modes (black arrows) is prohibitively slow in unbiased molecular dynamics simulation of this strongly charged complex. We therefore devised an alchemical thermodynamic cycle that couples an artificial state (dashed box) to the unmodified (‘native’) physical ensemble. The nonphysical analogue ATP^2.5–^·Mg^0.5+^ defined therein, with cation charge reduced to +0.5 and a compensating +1.5 charge spread uniformly over the atoms of the triphosphate, rapidly samples diverse Mg^2+^–phosphate configurations. By means of alchemical charge-scaling free energy calculations, we obtained the respective free energy differences (along green arrows) between multiple native subensembles, each restrained to a defined coordination mode, and this rapidly exchanging common reference state. These calculated free energy differences also define the relative free energies (along black arrows) between coordination modes in the unmodified force field.

The strength of the short-range electrostatic interactions between the divalent cation and the phosphate oxygen atoms leads the metal–phosphate group to adopt discrete tightly bound states, with two main distinct ATP^4–^·Mg^2+^ coordination modes in solution.^3,4^ These are defined by their coordination of the Mg^2+^ ion by either all three phosphate groups (tridentate coordination, here abbreviated as C3; Figure 1a, left) or by the terminal β and γ phosphate groups only (bidentate, C2; Figure 1a, right). Nuclear magnetic resonance,^3,5^ as well as infrared and Raman spectroscopy,^4^ detected both C2 and C3 coordination, with tentative support for an excess of C2, suggesting a small free energy difference of at most a few *k*_B_*T* between C2 and C3 in solution.

Both configurations are biochemically relevant, and the Protein Data Bank (PDB) contains many complexes in both C2 and C3 configurations. Both are observed as bound substrates of ATP-consuming enzymes, consistent with quantum chemical studies of solvated ATP·Mg^2+^,^6,7^ which estimated similar hydrolysis transition state free energies for C2- and C3-like configurations, respectively.

Computational studies of Mg^2+^–triphosphate coordination in solution similarly indicate both states are energetically accessible, notwithstanding the well-known challenges posed by strong charges and metal ions in atomistic simulations.^8^ Employing the CHARMM force field description of ATP·Mg^2+^,^9^ Liao et al.^10^ calculated a free energy difference between C2 and C3 close to zero, while Branduardi et al.^11^ reported a 2.9 kcal/mol preference for the C2 state. Although the transition between C2 and C3 involves only relatively small geometrical changes, both studies documented energy barriers high enough to preclude adequate sampling of coordination state transitions in unbiased molecular simulations. We are not aware of similar studies for other force fields, such as the ATP·Mg^2+^ Amber force field-compatible parametrization of Meagher et al.^12^

Here we assess the accuracy of force field descriptions of ATP·Mg^2+^, focusing on the configurational equilibria of Mg^2+^–phosphate coordination both in solution and bound to proteins. Applying an enhanced sampling protocol, we found substantial discrepancies, both between Amber and CHARMM force fields as well as with experiment. To resolve the disagreement, we combined the distribution of ATP·Mg^2+^ configurations in the PDB and configurational free energy calculations to derive quantitative corrections for both force fields.

## Methods

### Force Fields

We examined two force field representations of ATP·Mg^2+^. The CHARMM22 force field parameters^9^ are those employed in previous studies directly addressing ATP–Mg^2+^ coordination.^10,11^ As described below, we separately evaluated the CHARMM angle and dihedral parameter modifications derived by Komuro et al.,^13^ listed in Tables 1 and 2 therein. For the Amber force field, we used the parametrization of Meagher et al.,^12^ which is provided through a developer-endorsed collection of contributed parameters and has been widely adopted in combination with this force field.^14−18^

### Selection of PDB Reference Structures

Aiming to collect all instances of ATP and analogues with exactly one coordinated Mg^2+^ ion, we first retrieved all PDB structures containing both Mg^2+^ and ATP. From this selection, we discarded instances with more than one divalent cation within 8 Å of the ATP molecule and retained those with one Mg^2+^ ion within 3 Å of any phosphate oxygen. Redundant entries, as commonly arise from identical or homologous proteins or binding motifs, or multiple equivalent binding sites within crystal structures, were not removed, because they provide additional information on the structural flexibility. We thus obtained a sample of 2123 biomolecule-complexed configurations of ATP·Mg^2+^.

To aid spatial analysis of Mg^2+^–phosphate configurations, we performed a principal component analysis (PCA) using the coordinates of only the α-, β-, and γ-phosphate atoms, their two bridging oxygen atoms, and the Mg^2+^ ion, on the PDB data set. By averaging over the remaining configurational degrees of freedom, this analysis captures Mg^2+^–triphosphate coordination variance independently of the configurational diversity of the nucleotide.

### Unbiased Simulations

For an initial estimate of the rate of spontaneous coordination state transitions, we counted instances of simulations departing from their initial coordination mode in 200 unbiased molecular dynamics simulations, each of 1 μs length, comprising 50 simulations each starting from C2 and C3 configurations, under both Amber and CHARMM. The starting structures for these simulations were chosen from the PDB set for maximal configurational diversity, by maximizing the sum of mutual distances in the PCA space defined above (Supplementary S1).

### Enhanced Sampling Protocol

With unbiased simulations showing prohibitively slow exchange, we implemented an enhanced sampling approach to calculate free energy differences between coordination states. To this end, we devised an alchemical thermodynamic cycle that introduces a nonphysical, partially discharged intermediate, ATP^2.5–^·Mg^0.5+^ (Figure 1b, dashed box). With the cation charge reduction from +2 to +0.5 counterbalanced by a charge of +1.5 spread uniformly over the 13 phosphorus and oxygen atoms that comprise the triphosphate group, we maintain the same net charge while greatly reducing the height of the electrostatic barriers that impede sampling.

To couple this nonphysical alchemical state,^19^ designed for rapid configurational sampling, to the native force field ensembles of physical ATP^4–^·Mg^2+^, we defined a thermodynamic cycle spanning the reduced-charge and native states, interspersed by a set of mutually overlapping intermediate states (Figure 1b). Simulating this alchemical extended ensemble,^20^ we applied Hamiltonian Replica Exchange^21^ (HREX) to enable propagation of diverse configurations from rapidly exchanging reduced-charge states to the native state with correct thermodynamic weighting, thereby greatly accelerating sampling of the native ensemble.

To enforce sampling of higher-energy configurational regions of interest, we further defined 18 geometrically restrained native (ATP^4–^·Mg^2+^) subensembles, respectively confined to seven permutations of single, double, and triple coordination of Mg^2+^ to the α-, β-, and γ-phosphate groups, and to a further 11 barrier regions of interest. Each restrained state was coupled to the single common unrestrained reduced-charge state by a chain of 20 intermediates, spaced to equalize mutual phase space overlap along the chain as previously described,^22^ yielding average acceptance rates of 0.5 and regular end-to-end exchanges of configurations. The corresponding set of subensembles is enumerated in Supplementary S4, along with further simulation details.

To calculate free energy differences across all states of the extended ensemble, we applied the Multistate Bennett Acceptance Ratio^23^ (MBAR) estimator. In addition, we applied MBAR to reweight configurational samples collected across all subensembles of differing restraint potential and charge scaling to their correct thermodynamic weighting in the unrestrained, fully charged native ensemble. This protocol yields broad sampling covering both free energy minima and selected regions of higher-energy configurational space, from which arbitrary observables of interest may be extracted.

### Protein:ATP Free Energy Calculations

To calibrate force field free energies against the wealth of additional structural data points available from the PDB, we calculated free energy differences between coordination states for a set of 30 ATP·Mg^2+^–protein complex structures. Selecting these structures, we excluded proteins larger than 250 kDa, structures containing nonstandard protein residues or nonprotein cofactors (including additional metal ions), and structures with protonatable side chains in the ATP·Mg^2+^ binding region whose protonation state could not confidently be assigned. The otherwise arbitrarily selected set contained 17 C3 and 13 C2 complexes. Configurational free energies were calculated analogously to the solution protocol detailed above, with use of the same artificial ATP^2.5–^·Mg^0.5+^ intermediate, but incorporating only two restrained native (ATP^4–^·Mg^2+^) end states corresponding to C2 and C3 coordination. Further simulation details are reported in Supplementary S3.

## Results and Discussion

### Unbiased C2/C3 Transitions in Molecular Dynamics Simulation

Despite the simple geometry and small structural difference between the C2/C3Mg^2+^–phosphate configurations, the high intervening electrostatic energy barrier^11,24^ renders sampling of the equilibrium in unbiased simulation quite challenging. Indeed, in 200 unbiased 1-μs simulations as detailed under Methods, only seven transitions were observed, and those only for the CHARMM force field in the direction C3 → C2. No C2 → C3 transitions were seen under CHARMM, and no transitions were seen in either direction under Amber, highlighting the need for enhanced sampling approaches.

### Free Energy of C2/C3 Coordination Modes

**Figure 2:**
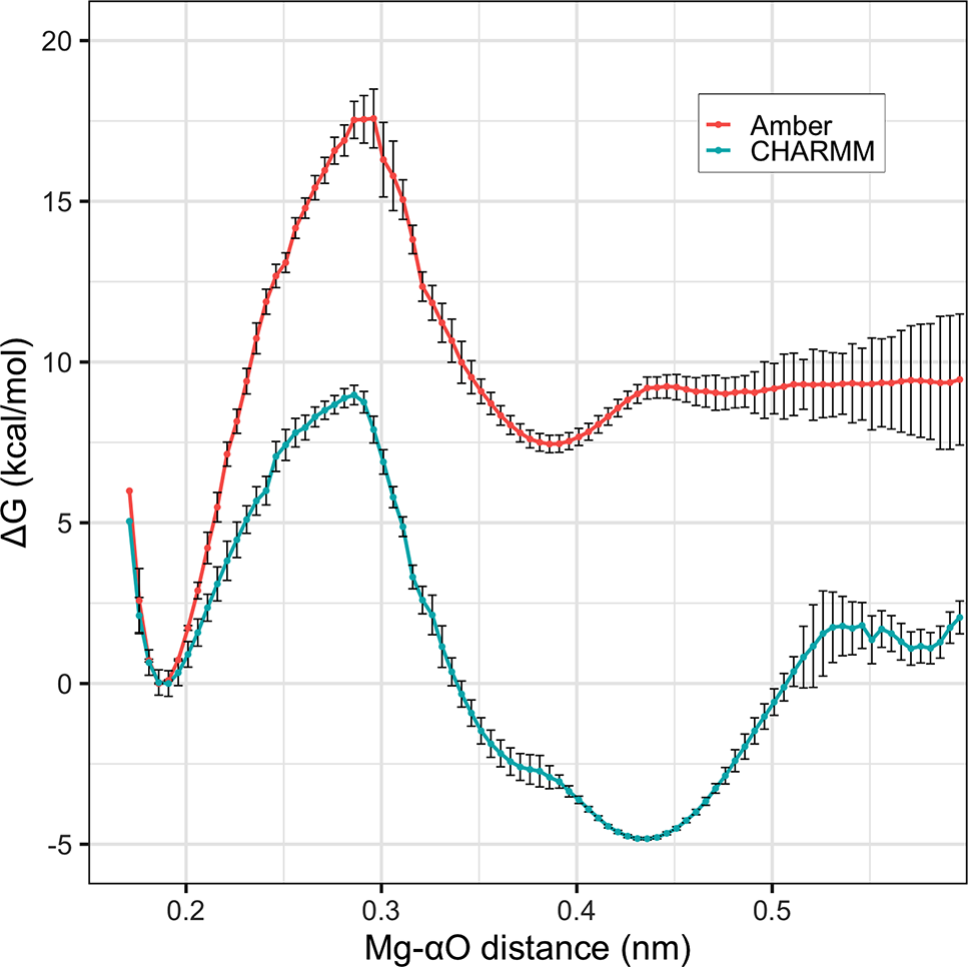
Free energy as a function of the distance between Mg^2+^ and the closest α-phosphate oxygen atom for Amber (red) and CHARMM (blue) parameter sets.

Applying the RE/MBAR enhanced sampling protocol (see Methods), we first assessed force field predictions of the C2/C3 equilibrium. Figure 2 shows the free energy as a function of distance from the Mg^2+^ ion to the nearest α-phosphate–oxygen distance (Mg−αO), revealing a substantial disagreement between the two force fields. Where experimental data suggest a slight preference for C2 over C3,^4^ the Amber ensemble shows C2 disfavored by 6.4 ± 0.1 kcal/mol, while CHARMM shows C2 favored by 5.3 ± 0.2 kcal/mol. On this measure, the two force fields thus disagree by a remarkable 11.7 ± 0.2 kcal/mol and predict opposing trends.

### Comparison of Free Energy Landscapes and PDB Configurations

To reconcile this discrepancy, and in the absence of further experimental data for solvated ATP·Mg^2+^, we next used ATP·Mg^2+^ configurations from the PDB as an additional source of information about the actual free energy difference between the C2 and C3 configurations. To this end, we initially assumed that this sample of ATP·Mg^2+^ configurations, although exposed to quite different surroundings compared to the solution ensemble, is governed by the same strong intracomplex electrostatic interactions as in solution and should thus exhibit similar configurations.

To test if this is actually the case, we examined whether regions of configurational space most densely populated with PDB structures overlap with low free energy regions observed in the simulations. The raw count of C2 and C3 ATP·Mg^2+^ instances in the PDB here provides initial support: of the 2123 retrieved configurations, 77.2% are found in the C2 (β–γ) and C3 (α–β–γ) coordination states, with 49.7% C2 and 27.5% C3, which is indeed consistent with the experimental finding that these forms predominate in solution and are approximately isoenergetic. The populations of all observed forms are enumerated in Supplementary S5.

**Figure 3:**
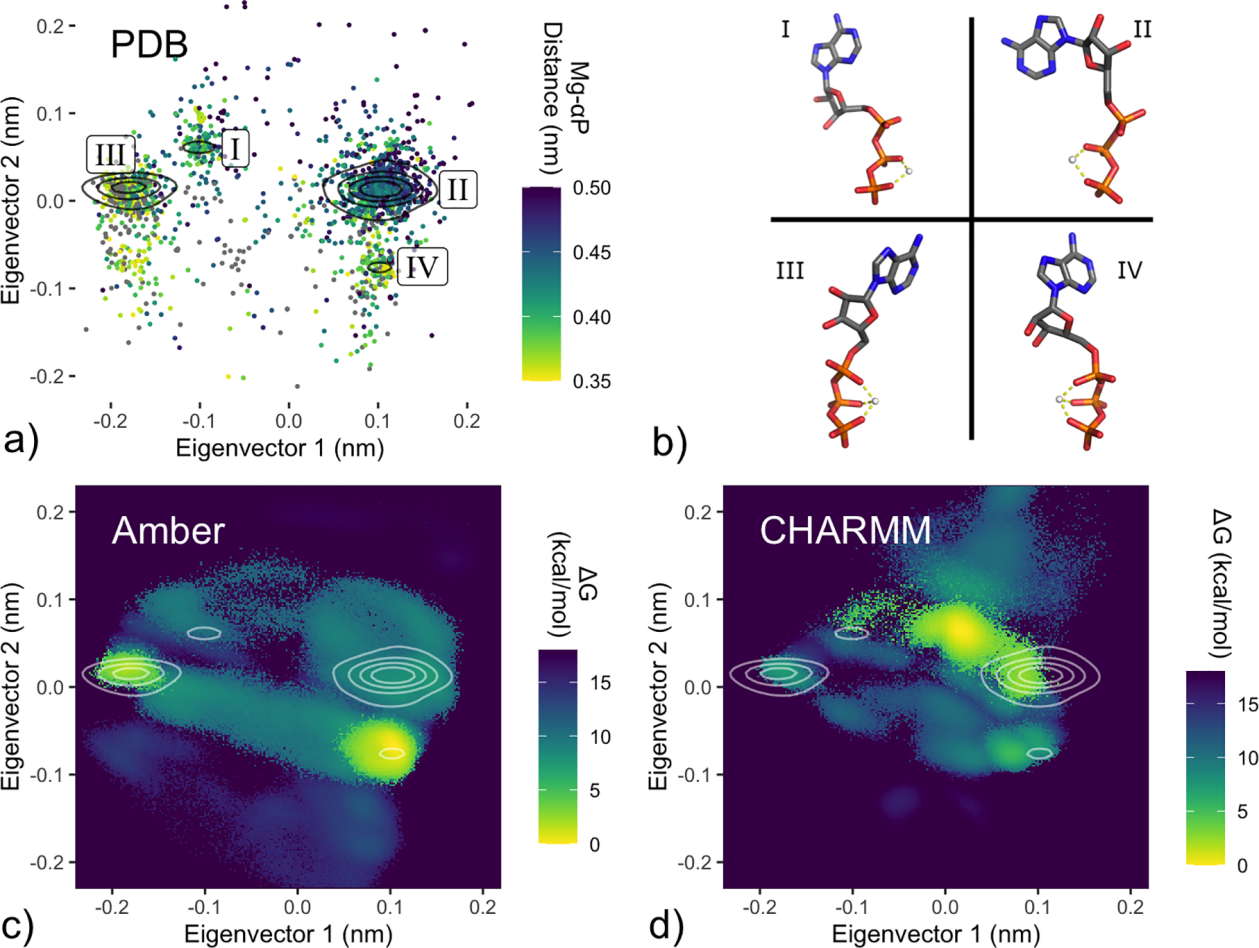
PCA projections of PDB configurations and force field free energy landscapes. a), c), and d) Projections of the first two eigenvectors of a principal component analysis incorporating six atoms of the Mg^2+^–phosphate tail (Pα, O3α, Pβ, O3β, Pγ, and Mg^2+^) as detailed under Methods. a) Distribution of PDB configurations. Points color-coded by the Pα–Mg^2+^ distance, where a distance below 0.375 nm corresponds to C2 coordination. Density estimate represented by black contour lines, with four identified clusters labeled I–IV. b) Representative configurations from labeled clusters I–IV. c), d) HREX/MBAR-derived solution free energies from Amber (c) and CHARMM (d) ATP·Mg^2+^ force fields, with the PDB density contours defined in panel a) shown in white.

A more detailed picture is provided by a PCA projection (Figure 3a) of the PDB set (detailed under Methods). This projection clearly separates C2 and C3 coordination (along the second eigenvector). In addition, the first eigenvector captures an inversion around the axis of the triphosphate tail (Figure 3b) for both coordination modes, yielding four rather than two main density clusters. Accordingly, labeled clusters I+II and III+IV, respectively, correspond largely to the C2 and C3 configurations.

To compare the simulated solution ensembles with the PDB set (Figure 3a), we represented their respective free energy landscapes under the same PCA projection. If the underlying configurational energy landscapes are similar, we would expect overlap between density of PDB structures and regions of low free energy in the simulated solution ensembles. Such overlap is indeed apparent in the projections of the Amber (Figure 3c) and CHARMM (Figure 3d) ensembles, with both force fields showing energetically favorable regions largely overlapping with PDB density. In particular, both show local minima corresponding closely to labeled PDB clusters III and IV. Regions more sparsely populated with PDB structures broadly show accessible free energy in the force field ensembles, and regions containing no PDB structures indeed correspond to regions of high energy in the simulated solution ensembles.

Beyond these aspects of qualitative agreement, the comparison reaffirms our finding that Amber disfavors the C2 subform (clusters I and II). This contradicts the CHARMM force field and is inconsistent with the PDB set, unless the majority of biomolecular complexes was to select a coordination mode that is substantially disfavored in solution, which is unlikely.

Further, the CHARMM ensemble shows a free energy minimum between clusters I and II of the PCA projection, which is absent in the Amber landscape and barely represented among PDB Configurations. Again, this result would imply that, for the CHARMM force field, the most favorable configuration in solution almost never occurs among known biomolecular complexes. Additionally, a substantial fraction of the most-populated PDB cluster II (eigenvector 1 > 0.1) is assessed to be energetically inaccessible (>+18 kcal/mol) in this projection of the solution free energy landscape, contradicting the Amber force field and implying that PDB structures observed in this region must overcome a large energy penalty.

Notwithstanding the different energetics expected for solvent and protein environments, the above disparities between the simulated solution ensembles and the protein-complexed PDB set are concerning. Further, the outright contradictions between the two simulated solution ensembles show that one or even both force fields describe the energetics, and possibly the structures, of the C2 and C3 coordination states inaccurately.

### Free Energy Calculations for Experimental ATP–Protein Complexes

For a closer, quantitative assessment of the energetics of the two ATP·Mg^2+^ configurations for both force fields, we next harnessed the diversity of biomolecular surroundings in the PDB sample. Because each PDB structure represents an energetic minimum of an ATP–protein complex, a free energy calculation comparing the two configurations should, with an accurate force field, always evaluate the experimentally observed coordination state as lower in free energy than the alternative state. Applied to a set of complexes sufficiently large to sample a broad range of free energy differences between C2 and C3 configurations, this principle permits measurements of force field accuracy and, in turn, quantification of the systematic mispredictions that the above analysis strongly suggests. In contrast to the qualitative analysis above, this approach does not assume direct comparability of the solution ensemble and the PDB distribution.

**Figure 4:**
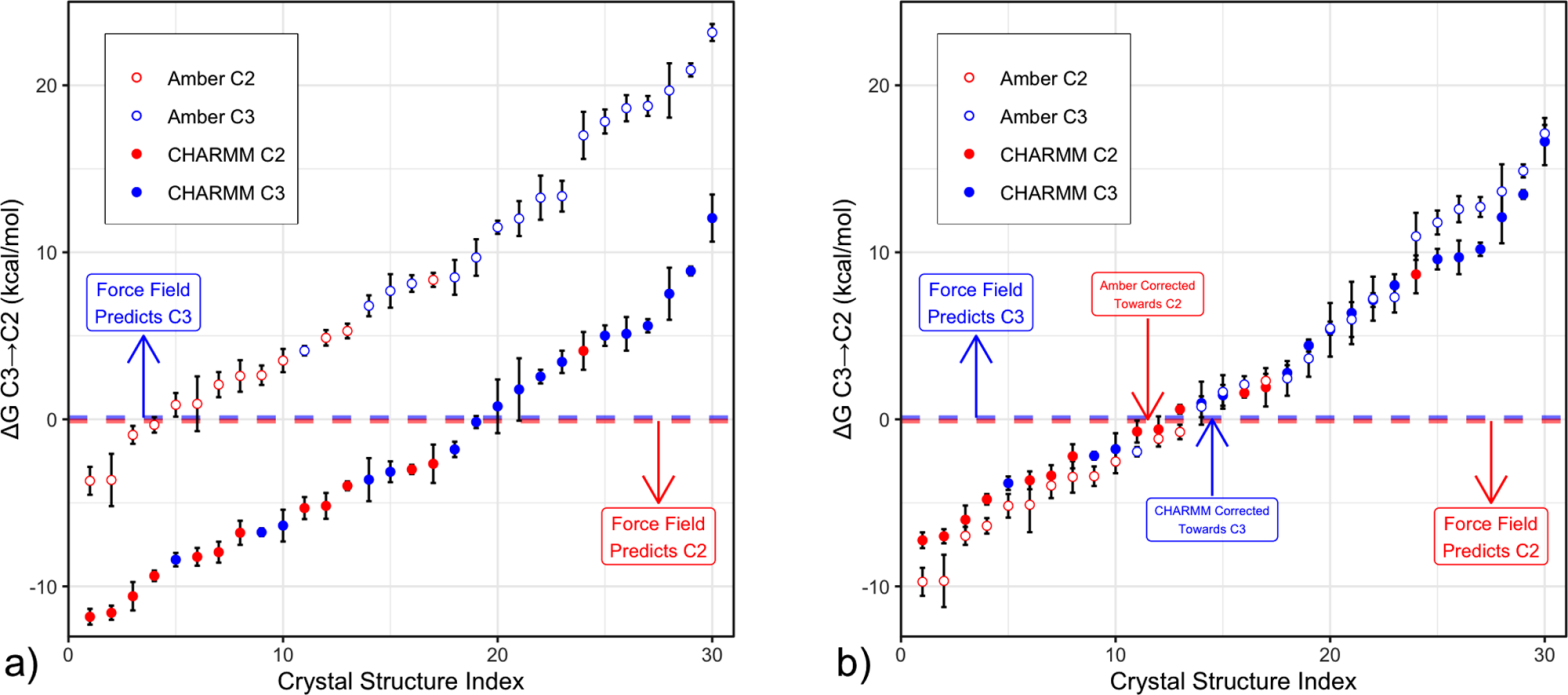
**a)** Calculated free energy difference for the transition from C3 to C2 coordination for a set of 30 biomolecular complexes of ATP·Mg^2+^, using Amber (open circles) and CHARMM (filled circles) force fields, with C2 crystal structures colored red and C3 crystal structures colored blue. A perfectly accurate force field would place all red points below zero and all blue points above zero. Both force fields display substantial predictive power, with red points more negative and blue points more positive, but absolute values are systematically biased in opposite directions. **b)** Respective linear offsets to the C2/C3 configurational free energy for each force field counteract this bias, achieving absolute agreement (within sampling uncertainty) for 28 of 30 instances with the Amber force field and 24 of 30 instances with CHARMM.

To this end, we calculated the free energy difference between C2 and C3 configurations using both force fields for a selected set of 30 ATP·Mg^2+^–protein complexes, as described under Methods. In Figure 4a, the color indicates which configuration is actually seen in the PDB (blue: C3; red: C2), whereas the force field prediction is indicated by the sign of the obtained free energy difference (positive: force field predicts C3; negative: C2). Hence, an accurate force field should place all blue points above the dashed zero line and all red points below.

Overall, both CHARMM (filled circles) and Amber (open circles) are able to differentiate the coordination mode in most instances, with C3 (blue) points mostly showing a more positive free energy difference than the C2 (red) ones. Indeed, for Amber, 16 of the 17 most positive free energy values correspond to C3 PDB structures, and 12 of the 13 most negative correspond to C2 PDB structures. Likewise for CHARMM, 14 of the 17 most positive values are C3, and 10 of the 13 most negative are C2.

However, the calculated free energies from both force fields are offset both with respect to each other as well as to the zero free energy line, in accordance with our observation above for the solution free energies. Amber systematically mispredicts C3 coordination for C2 PDB structures, with only 4 of 13 C2 structures correctly predicted, whereas CHARMM appears biased toward C2, predicting only 10 of 17 C3 structures correctly.

The very good relative performance of both force fields suggests a simple additive correction that can be extracted from the calculated free energies of the selected protein complexes by shifting both sets of free energy values up or down, as shown in Figure 4b, to optimally meet the positive/negative sign criterion and thus to maximize agreement with PDB observations. For the Amber force field, applying an offset of between −7.1 and −5.1 kcal/mol yields predictions consistent with the PDB structure for 28 of 30 members of the test set. Using the mean value of −6.1 kcal/mol for the correction, all but two binding modes are predicted correctly, and the two remaining mispredictions deviate by less than ±2.0 kcal/mol from a correct prediction. For CHARMM, an offset in the range +2.5 to +3.3 kcal/mol yields a prediction consistent with experiment for 24 of the 30 tested complexes, and for the mean value of +2.9 kcal/mol, violations between −2.5 and 5.9 kcal/mol are observed for the remaining four incorrect predictions.

### Reevaluation of Solution Energy Landscapes

Next, we asked if these force field corrections also improve the description of the C2/C3 population in solution. The unmodified force fields predicted C3 → C2 free energy differences in solution of +6.4 ± 0.1 kcal/mol (Amber) and −5.3 ± 0.2 kcal/mol (CHARMM). Applying the respective lower and upper bounds of the offset ranges determined above (−7.1 and +3.3 kcal/mol), and with the assumption that these offsets, derived in a diverse set of partially solvated biomolecular environments, are applicable to condensed-phase simulations in general, these differences reduce to −0.7 ± 0.1 kcal/mol (Amber) and −2.0 ± 0.2 kcal/mol (CHARMM). Within sampling and experimental uncertainty, these free energy differences are consistent with the populations observed by NMR. Considering the large initial disagreement (11.7 ± 0.2 kcal/mol) and, more broadly, the challenges inherent to force field simulation of strongly charged chemical entities and metal ions, we consider this result remarkably accurate.

**Figure 5:**
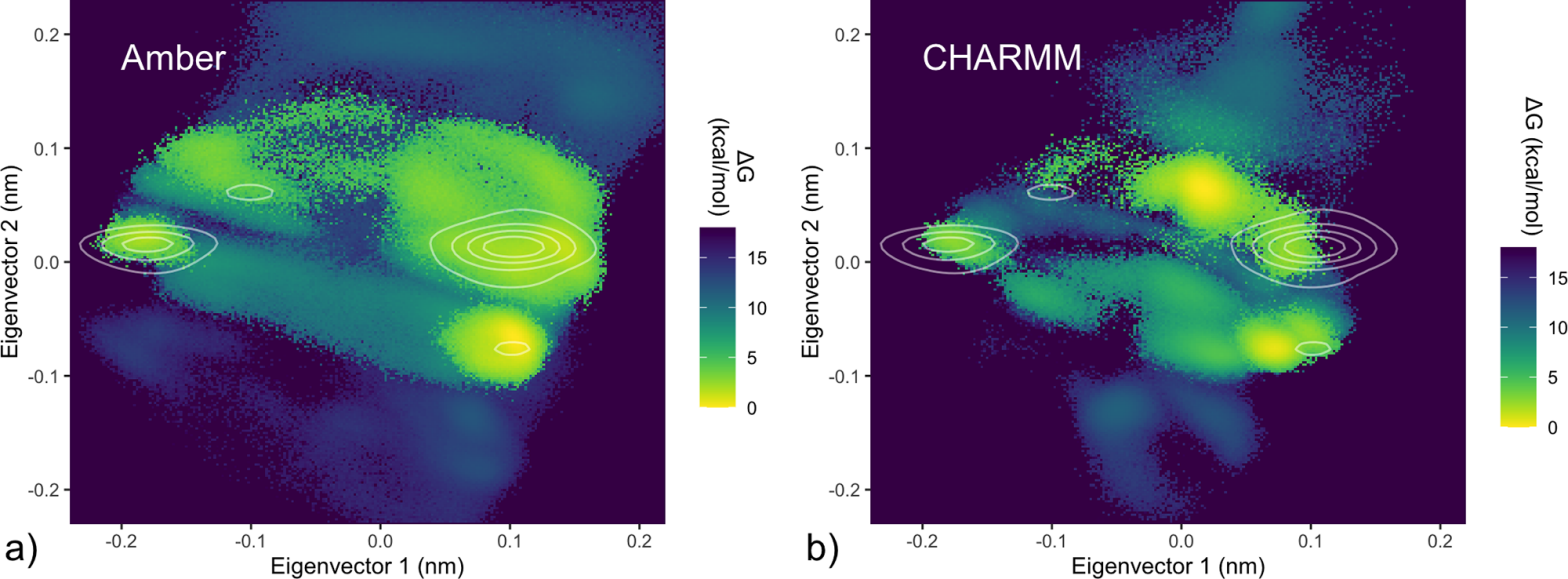
Reweighted free energy maps. Solution free energy landscapes using the same eigenvector projection as Figure 3, for Amber (a) and CHARMM (b) force fields, reweighted according to the described C2/C3 corrections; PDB clusters shown as contour lines. Postcorrection, C2 and C3 configurations (upper and lower clusters respectively; Figure 3b) are approximately isoenergetic, in agreement with experiment.

Further, we asked if these corrections also produce structural ensembles that better agree with the PDB sample. To this end, we applied the respective C2/C3 offsets as weighting factors to all C2 frames (defined by a Mg^2+^–αP distance greater than 3.75 Å) of each force field ensemble, yielding revised solution free energy landscapes for each force field. Figure 5 depicts these reweighted energy landscapes, using the same PCA projection as in Figure 3.

Indeed, for the corrected Amber free energy landscape (Figure 5a), agreement is improved, with defined minima closely matching areas of PDB configurational density, and the previous disfavoring of C2 structures (clusters I and II) is no longer seen.

Similarly, the corresponding corrected CHARMM free energy map (Figure 5b) reflects a reduced C2 preference. However, the deepest free energy minimum still lies in a region of configurational space barely represented in the PDB set. Further, a substantial fraction of configurations in PDB cluster II (eigenvector 1 projection >0.1 nm) is evaluated to be energetically inaccessible in solution (>18 kcal/mol).

**Figure 6:**
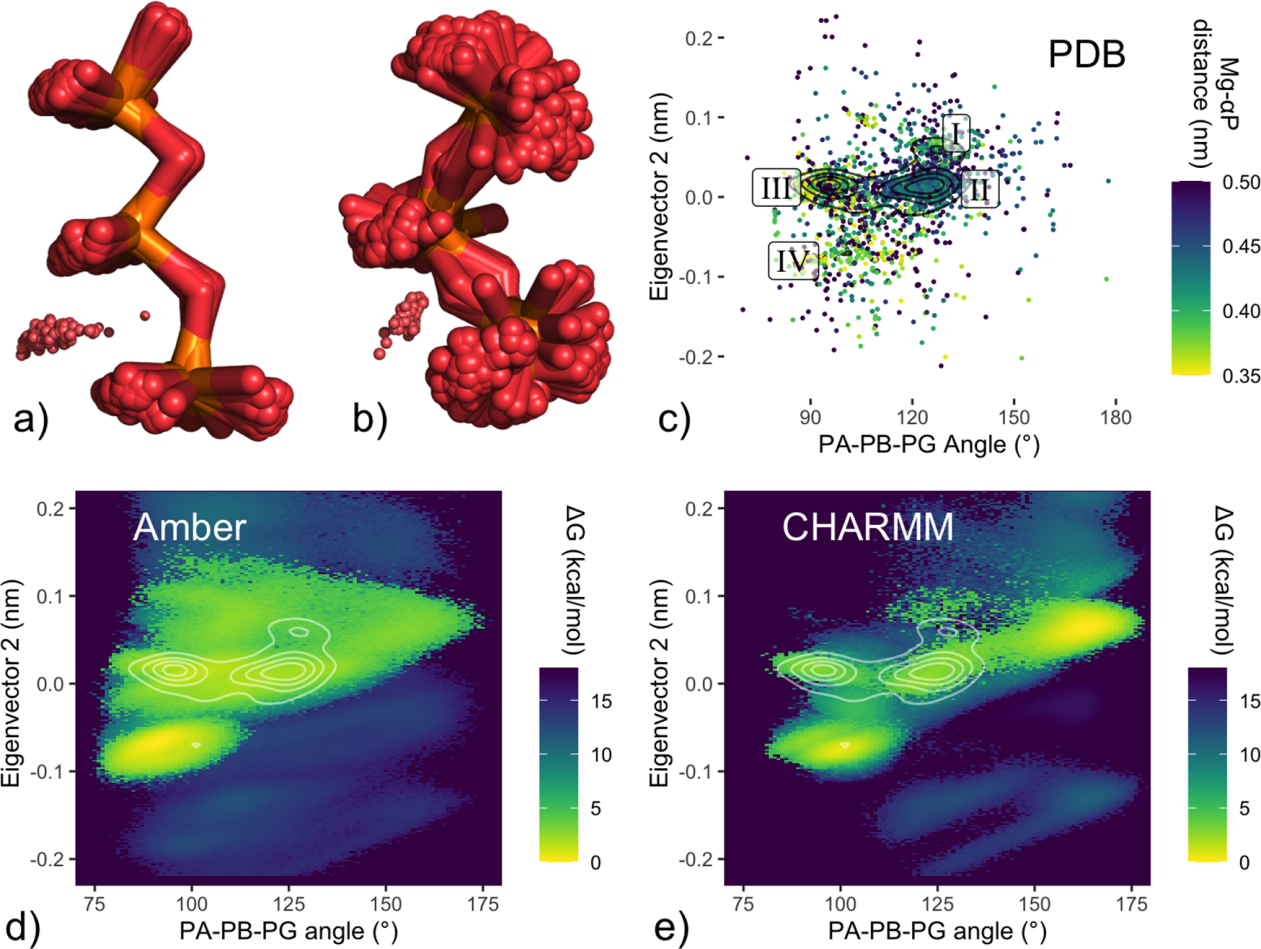
Overextended phosphate configurations under CHARMM. **a)** Structures from CHARMM energy minimum not found in PDB. Overlay of the 100 lowest-energy MD structures, which occur in the region with eigenvector projection [EV1 = 0.02, EV2 = 0.06] (Figure 5b), between labeled clusters I and II, which is only sparsely populated among the PDB data set. These structures show an extended triphosphate configuration, with a mean α–β–γ-phosphate angle of 161°. **b)** PDB structures, inaccessible under CHARMM: overlay of 100 randomly selected triphosphate configurations from the PDB with projections [EV1 > 0.11 nm, −0.02 nm < EV2 < 0.04 nm], corresponding to the region of PDB cluster II that is energetically inaccessible under the CHARMM force field (Figure 5b); these structures show a compact triphosphate configuration (mean α–β–γ-phosphate angle of 112°). **c)–e)** Reprojections as a function of the α–β–γ-phosphate angle for PDB configurations (c) and of free energy landscapes (reweighted as described for Figure 5) under Amber (d) and CHARMM (e). e) shows the CHARMM minimum extending beyond 150°, while PDB structures (c) are barely observed in this region. The Amber landscape (d) shows extended configurations, beyond 130°, as energetically accessible but less favorable.

Investigating the geometric basis of this discrepancy under the CHARMM force field, Figure 6a shows an overlay of the 100 lowest-energy configurations within the CHARMM minimum between clusters I and II. Examination of this set reveals β–γ-coordinated (C2) structures with extended phosphate tail configurations, with a mean value of 161° for the α–β–γ-phosphate angle. Conversely, PDB configurations from the region of cluster II that is energetically inaccessible under CHARMM (Figure 6b) display a less-extended phosphate tail, with an average α–β–γ-phosphate angle of 112°.

Both observations suggest that the CHARMM force field favors extended phosphate tail configurations. To test this hypothesis, we reprojected the simulated free energy landscapes as a function of the α–β–γ-phosphate angle. This representation confirms a clear separation of the CHARMM minimum beyond 150° (Figure 6e), deviating from the PDB distribution, which is barely populated beyond 135° (Figure 6c). By contrast, the same projection of the Amber ensemble (Figure 6d) shows a region of extended-phosphate configurational space that is accessible, but disfavored by >3 kcal/mol, along with good overall agreement between the free energy landscape and the PDB distribution.

Indeed, the same property was already apparent for the free energy as a function of the Oα–Mg^2+^ distance: in Figure 2, the CHARMM trace displays a broad C2 subminimum for extensions beyond 4.2 Å, whereas the Amber minimum at 3.8 Å is narrower. More generally, the geometry of tridentate Mg^2+^-*αβγ* coordination dictates a more folded tail configuration for C3. These observations suggest that the CHARMM force field preference for more extended configurations may also explain its relative disfavoring of C3.

Taken together, our results show that CHARMM favors overextended configurations that are barely represented among known biomolecular complex configurations, while evaluating a substantial fraction of experimentally observed, less-extended C2 PDB configurations as energetically inaccessible. In isolation, this observation would not contradict reported spectroscopic measurements that suggest a preference for β–γ coordination. Likewise, the disparity between regions of low free energy in solution and the most commonly observed PDB structures alone would not rule out the possibility that particular configurational regions are populated in solution but not among biomolecular complexes or vice versa. However, the close agreement between the reweighted Amber ensemble and the PDB distribution is further evidence against this already implausible interpretation, rendering it quite likely that the identified overextended CHARMM minimum is unrealistic.

**Figure 7:**
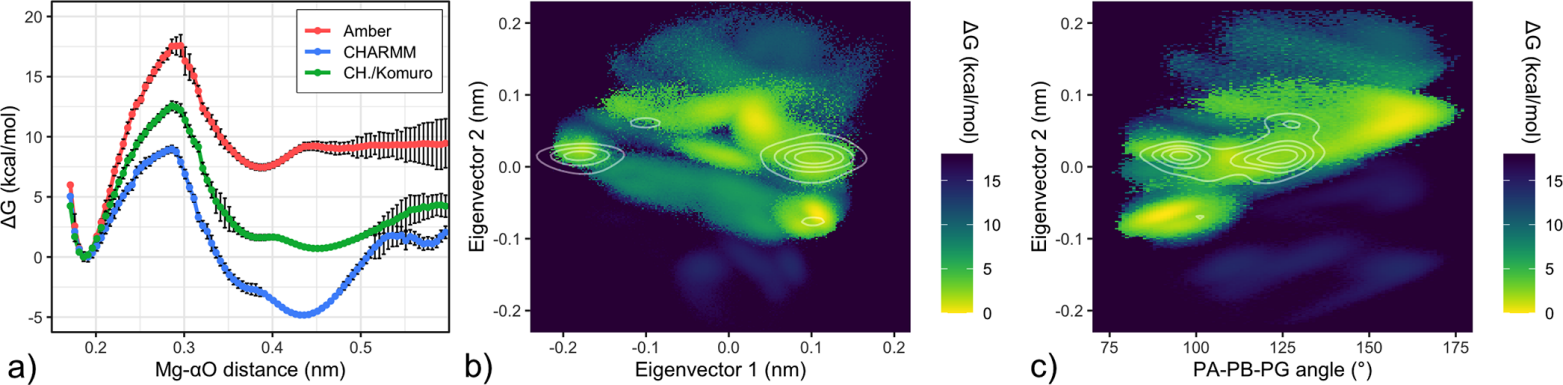
CHARMM reparametrization of Komuro et al. (2014), designed to rectify triphosphate overextension. **a)** Free energy as a function of the OA–Mg^2+^ distance (Komuro et al. reparametrization in green) shows C2 and C3 states close to isoenergetic, while retaining the previously noted subminimum extending beyond 4.2 Å. **b)** PCA projection of the free energy landscape for the Komuro et al. CHARMM parameters, analogous to the CHARMM ensemble in Figure 5b. While the right-side region of PDB density cluster II is no longer evaluated to be energetically inaccessible, and low free energy regions align well with all four marked clusters, we note that a region of low free energy centered around EV1 = 0, which is barely populated among PDB structures, persists. **c)** Free energy as a function of the α–β–γ-phosphate angle and eigenvector 2, as shown in Figure 6e for unmodified CHARMM. As in panel b), while agreement with PDB density clusters is markedly improved, the extended form (α–β–γ-phosphate angle > 150°) persists as a free energy minimum.

This finding agrees with that of Liao et al.,^10^ that crystallized ATP configurations appear elastically strained under CHARMM. Also, Komuro et al.^13^ noted a bias toward triphosphate overextension in CHARMM simulations of selected protein–ATP complexes, leading to an inability to reproduce crystallized configurations. In response, the authors conducted a partial reparametrization of the triphosphate angle and dihedral parameters, which did allow the crystal configurations in question to be reproduced. We implemented this force field modification and evaluated it with our RE/MBAR protocol (Figure 7).

As can be seen, the C2/C3 free energy now agrees well both with the corrected force field consensus and with experiment, with a marginal −0.32 ± 0.10 kcal/mol preference in favor of C2. Further, the PDB density clusters agree well with low free energy regions in this simulated solution ensemble. The overextension that motivated the reparametrization appears partially mitigated, though not eliminated, with the energy minimum between PDB clusters I and II (Figure 7b) and at greater phosphate extension (Figure 7c) still present, disagreeing with the PDB set and the Amber force field.

Separately, we examined the Mg^2+^ reparametrization of Allnér et al.,^8^ which addressed the unphysically slow hydration shell and nucleic acid exchange rates. As detailed and discussed in Supplementary S7, we found only minor effects on the C2/C3 free energy difference and no substantial impact on agreement between the respective force field free energy landscapes and the distribution of PDB structures.

## Conclusions

Our accuracy assessment of two broadly applied force field descriptions of ATP·Mg^2+^, focusing initially on the configurational equilibrium between C2 (bidentate) and C3 (tridentate) coordination, revealed a pronounced disagreement between the two parameter sets. CHARMM and Amber each predicted substantial preferences in opposite directions, with neither reproducing the experimental finding that these configurations are close to isoenergetic in solution.

Addressing this discrepancy, we compared simulated solution free energy landscapes to the distribution of ATP·Mg^2+^ configurations found among biomolecular complexes in the PDB. Configurational free energy calculations for 30 structurally diverse ATP–protein complexes served to quantify systematic force field biases that led to mispredictions of the PDB coordination mode and enabled corrections to the relative free energy of the C2 and C3 coordination modes for each force field.

For the Amber force field, our subsequent evaluation showed an encouraging degree of overlap between regions of low free energy in solution and the most common configurations observed among protein complexes in the PDB, suggesting that the underlying physical and simulated energy landscapes are similar. For biomolecular simulations in which only a single ATP·Mg^2+^ coordination mode needs to be sampled, no correction of the C2/C3 equilibrium is required. In rarer cases where this criterion is not fulfilled, our correction and the resulting reweighting enable a straightforward *a posteriori* correction of the sampled ensembles, without much additional computational effort. The enhanced sampling protocol we described presents a robust solution to sampling across the high electrostatic barriers that separate coordination states.

By contrast, we observed a pronounced tendency toward overextension of the triphosphate group under CHARMM, in line with earlier observations.^10,13^ This extended configuration is separated from the more compact C2 subconfigurations found in the PDB only by low energy barriers, which can readily be crossed in equilibrium simulations. Contrasting with the high energy barriers that separate C3 configurations from this overextended region, which permit equilibrium sampling under CHARMM of a C3 region that broadly reflects the distribution of configurations found in the PDB, CHARMM ensembles of C2 ATP·Mg^2+^ will thus sample a region that matches neither the PDB nor the predictions of the Amber force field. The force field modifications of Komuro et al.^13^ alleviate but do not eliminate this tendency.

Underscoring the importance of more accurate theoretical representations of both structure and energetics of ATP·Mg^2+^, C2 configurations comprise the majority of PDB structures. Accordingly, many typical biomolecular simulation applications incorporating ATP·Mg^2+^ will sample a distorted equilibrium under CHARMM. In particular, the single largest cluster in the PDB (Figure 3a, cluster II) is dominated by structures containing the prototypical P-loop ATPase motif, which mediates energy-consuming biochemical processes across domains of life^25^ and which binds ATP·Mg^2+^ in its C2 configuration.

Assuming that the free energy of any structural property observed in a PDB structure is lower than that of alternative configurations, we have demonstrated how the wealth of free energy information contained within the many available protein structures in the PDB can be harnessed to assess and improve biomolecular force fields. We think this approach is quite general and should be applicable to many other molecular properties for which experimental data are scarce or hard to obtain but where a diverse sample of instances can be obtained from the PDB.

## Supporting Information Available

S1, selection of diverse starting points for unbiased simulations; S2, symmetry-related configurations; S3, simulation details; S4, thermodynamic cycle and MBAR analysis of solution ensembles; S5, ATP·Mg^2+^ coordination modes observed in PDB; S6, protein free energy calculation results; and S7, Allnér et al. ion parameters (PDF)
