# Supplementary material for: ATP–Magnesium Coordination: Protein Structure-Based Force Field Evaluation and Corrections
Source: J Chem Theory Comput. 2021 Feb 22;17(3):1922–30. doi: 10.1021/acs.jctc.0c01205 (PMC8023659; doi:10.1021/acs.jctc.0c01205)
Supplement: Supplementary file 1 — ct0c01205_si_001.pdf [file ct0c01205_si_001.pdf]

**Supplementary Material**

**S1. Selection of diverse starting points for unbiased simulations:** as detailed under "Unbiased Simulations" in the Methods section, we selected 50 C2 and 50 C3 structures from the PDB data set as starting points for unbiased molecular dynamics simulations, with the aim of evaluating the frequency of spontaneous coordination mode transitions. These starting configurations were selected for maximal configurational diversity by maximising the mutual distance between chosen configurations under the PCA projection described.

To this end, we first randomly selected a 50-member candidate set from the PDB set, and then maximised the sum of mutual distances by attempting replacement of one randomly selected entry from the candidate set with one randomly selected from the PDB set, retaining the switch only if the sum of all pairwise distances increased, and iterating until convergence was reached. This process was performed separately for C2 and C3, yielding two 50-member sets of starting configurations with maximal mutual distance under the PCA projection.

**S2. Symmetry-related Configurations**

The PCA projection of PDB ATP.$Mg^{2+}$ configurations and the corresponding force field free energy landscapes (Figure 3) separates symmetry-related pairs for each coordination mode along the first principal axis, denoted Clusters I and II (C2 coordination), and Clusters III and IV (C3 coordination). These configurations are symmetrical with respect to the achiral triphosphate group with which the cation interacts directly, and the energy landscape of isolated an triphosphate-$Mg^{2+}$ complex would accordingly be symmetrical along this axis. However, the ribose sugar of ATP imposes chirality on the complex, yielding non-identical surroundings for each of the pairs, as can be observed in the projected force field energy landscapes.

In the presented PCA projection, clusters I and III (EV1 < 0) are defined by a Pα-O3α-O3β-$Mg^{2+}$ dihedral angle below zero, and II and IV (EV1 > 0) by an angle greater than zero.

**S3. Simulation Details**

Simulations were performed using GROMACS[1] version 2019.6. All simulations used the stochastic dynamics integrator, at 310K, with a 2fs time step. All bond lengths of the solute were constrained using LINCS with an expansion order of 4, and water geometry was constrained using the SETTLE algorithm. Electrostatic interactions were calculated with PME[2], employing a real-space cutoff of 10Å. A pressure of 1atm was maintained using the Parrinello-Rahman barostat.

**S3.1. Solution Simulations**

For solution simulations, we used GROMACS tools to define a periodic dodecahedron with a 10Å minimum distance from the ATP.$Mg^{2+}$ solute to the edge of the box. The simulated system included 1256 molecules of TIP3P water and two $Na^{+}$ ions. For each tested force field, a total 9μs of simulation data was collected, spread evenly across all transformations between the common reference state and each of the 18 restrained sub-ensembles (Section S.3). Each transformation consisted of 20 alchemical intermediates, yielding a total of 360 thermodynamic states per force field.

### S3.2. Protein Simulations

Our free energy calculation approach aimed to quantify ATP.$Mg^{2+}$ configurational free energy differences in a diverse sample of crystal surroundings. Since highly polar ATP.$Mg^{2+}$ generally binds in solvent-exposed cavities on the protein surface, the neglect of crystal contacts would in many cases substantially alter the binding environment that yielded the observed configuration. To avoid neglecting these interactions, we simulated cubic boxes extracted from the crystal lattice. To this end, for each PDB structure, we first generated symmetry neighbours adjoining the central unit cell using Pymol[3], and from the resulting partial crystal lattice extracted a cubic simulation box with an edge length of 50Å, with ATP.$Mg^{2+}$ at its center. Position restraints with a force constant of 600 kcal.$mol^{-1}$.$nm^{-2}$ were applied to all atoms of every residue with at least one atom within 3Å of the box edge. We added water to each system with an additional 3Å shell around the 50 cube extracted from the crystal lattice, yielding a cubic simulation system with an initial 56Å edge length. $Na^{+}$ and $Cl^{-}$ ions were added as required to neutralise each system and to yield a salt concentration of 150mM, calculated as a fraction of the number of water molecules in the system (0.15M NaCl / 55.6M $H_2O$ = 1 NaCl / 370 $H_2O$). Molecular dynamics simulation parameters were identical to those used for the solution simulations as described above. Simulation data was collected for at least 350ns per system (equally spread among the alchemical intermediates), of which the final 80% were included in the analysis.

### S4.1. Thermodynamic Cycle

As detailed under Methods and illustrated in Figure 1, we defined a thermodynamic cycle that linked multiple selected regions of configuration space, defined by distances between the $Mg^{2+}$ ion and the α, β and γ phosphate atoms as defined below, to a common reference ensemble without distance restraints but with atomic charges of the $Mg^{2+}$ and triphosphate group scaled to facilitate rapid configurational sampling.

To prevent dissociation of the $Mg^{2+}$ ion from ATP in partially discharged states, a flat-bottomed distance restraint with a force constant of $10^6$ kJ $mol^{-1}$ $nm^{-2}$ was applied to $Mg^{2+}$-β phosphate distances beyond 12Å in all simulations. While in practice never violated, this restraint also has the effect of disallowing $Mg^{2+}$-dissociated configurations for the fully-charged complex.

Distance restraints enforcing $Mg^{2+}$-phosphate distances were defined using the “restraint potential” bond function (GROMACS function type 10). We defined **C (**close), **F** (far) and **B** (barrier) restraints as follows:

| | low (nm) | $up_1$ (nm) | $up_2$ (nm) | $K^{dr}$ (kJ $mol^{-1}nm^{-2}$) |
|---|---|---|---|---|
| C | 0 | 0.34 | 0.5 | $10^5$ |
| F | 0.375 | 0.7 | 0.85 | $10^5$ |
| B1 | 0.375 | 0.375 | 0.5 | $10^6$ |
| B2 | 0.345 | 0.345 | 0.5 | $10^6$ |
| B3 | 0.365 | 0.365 | 0.5 | $10^6$ |
| B4 | 0.385 | 0.385 | 0.5 | $10^6$ |
| B5 | 0.395 | 0.395 | 0.5 | $10^6$ |

These restraint potentials were applied to $Mg^{2+}$-phosphate distances to define 18 configurationally restrained sub-ensembles as follows:

| | $Mg^{2+}$-Pα | $Mg^{2+}$-Pβ | $Mg^{2+}$-Pγ |
|---|---|---|---|
| 1 | C | C | C |
| 2 | F | C | C |
| 3 | C | F | C |
| 4 | C | C | F |
| 5 | C | F | F |
| 6 | F | C | F |
| 7 | F | F | C |
| 8 | B1 | B1 | B1 |
| 9 | - | B1 | B1 |
| 10 | B1 | - | B1 |
| 11 | B1 | B1 | - |
| 12 | B1 | - | - |
| 13 | - | B1 | - |
| 14 | - | - | B1 |

| | | | |
|---|---|---|---|
| 15 | B2 | - | - |
| 16 | B3 | - | - |
| 17 | B4 | - | - |
| 18 | B5 | - | - |

Each of these end states was coupled by an alchemical transformation pathway to the common reference ensemble (described in Methods and Figure 1: no distance restraints imposed, $Mg^{2+}$ and triphosphate charges reduced). Atomic charges and the respective restraint potentials were scaled along a series of 20 alchemical intermediate states, as described under Methods. The values of the scaling parameters (λ) for electrostatic and restraint components for each intermediate state along each of the 18 20-state pathways linking the reduced-charge common ensemble ensemble to the 18 states above are included as a supplementary text file.
ffre
We emphasise that, for the calculation of only the C2/C3 free energy difference that was our primary focus, sampling of restrained states 1 and 2 (αβ and αβγ coordination) is sufficient. For the protein calculations, we only calculated αβ / αβγ free energy differences, using only states 1 and 2. For the solution simulations, the remaining states, which describe minor coordination modes and selected intervening regions, served to sample energetically unfavourable regions that would not otherwise be represented. The particular emphasis on the barrier separating αβ from αβγ coordination (restraints B2-B5) served to aid comparison with sampling along the equivalent physical reaction coordinate presented in earlier work[4].

### S4.2. MBAR analysis of solution ensembles

Each of the 18 transformations described above was divided into 20 alchemical intermediate states, yielding a total of 360 thermodynamic states for the analysis. Each analysis (one per force field) incorporated a total of 19-28 million sampled data points. With MBAR summation scaling as $N^3$ for the number of states, iterative solution over the full 360-state extended ensemble is prohibitively slow. We therefore analysed each of the 18 20-state alchemical pathways linking the common reference ensemble to each restrained sub-ensemble (table, S3.1) separately, and used the converged free energy estimates thereby obtained as input for the full 360-state analysis. Free energy estimates and sample weights were then finalised by 10 further iterations over the full 360-state extended ensemble.

As an output of the MBAR analysis, we recorded the estimated thermodynamic weight for every input frame under a natively charged, unrestrained Hamiltonian, allowing the relative weights of states defined by geometric observables of choice to be determined by summation of weights belonging to each bin (main text, Figures 2, 3, 5, 6 and 7).

## S5. ATP.$Mg^{2+}$ coordination modes observed in pdb

Among the set of ATP.$Mg^{2+}$ configurations collected from the PDB as described under Methods, the proportions of each coordination mode, with a contact to α, β or γ phosphate defined as a distance <3Å between $Mg^{2+}$ and any oxygen of the phosphate group, were as follows:

| | α-β-γ (C3) | β-γ (C2) | α-β | α-γ | α | β | γ |
|---|---|---|---|---|---|---|---|
| % | 27.5 | 49.7 | 6.1 | 2.9 | 2.2 | 4.1 | 7.5 |

## S6. Protein free energy calculation results

The following table lists calculated configurational free energy differences for the C3→C2 transition. The column "C2/C3" designates the configuration observed in the PDB structure.

| PDB ID | Amber C3→C2 ΔG (kcal/mol) | CHARMM C3→C2 ΔG (kcal/mol) | C2/C3 |
|---|---|---|---|
| 1B38 | 20.9 ± 0.4 | 12.1 ± 1.4 | C3 |
| 1D4X | 0.9 ± 1.6 | -11.6 ± 0.4 | C2 |
| 1J09 | 13.4 ± 0.9 | 5.1 ± 1.0 | C3 |
| 1KO5 | 4.9 ± 0.5 | -3.0 ± 0.3 | C2 |
| 1OBD | 7.7 ± 1.0 | -1.8 ± 0.5 | C3 |
| 1TID | 18.8 ± 0.6 | 5.6 ± 0.4 | C3 |
| 1XEX | -3.7 ± 0.8 | -11.8 ± 0.5 | C2 |
| 1XMJ | 3.5 ± 0.7 | -2.7 ± 1.2 | C2 |
| 1Y8P | 23.2 ± 0.5 | 8.9 ± 0.3 | C3 |
| 1YID | 4.1 ± 0.3 | -6.8 ± 0.2 | C3 |
| 2A84 | 6.8 ± 0.6 | -0.2 ± 0.4 | C3 |
| 2BBT | 2.6 ± 0.9 | -5.2 ± 0.8 | C2 |
| 2OLR | 9.7 ± 1.1 | -8.4 ± 0.4 | C3 |
| 2PZF | -0.3 ± 0.5 | -8.2 ± 0.5 | C2 |
| 2RD5 | 17.8 ± 0.7 | 5.0 ± 0.6 | C3 |
| 2VHQ | 13.3 ± 1.3 | 0.8 ± 1.6 | C3 |
| 2W00 | 2.6 ± 0.6 | -4.0 ± 0.3 | C2 |
| 2Y27 | 5.3 ± 0.4 | 4.1 ± 1.1 | C2 |
| 2ZHZ | 19.7 ± 1.6 | 3.4 ± 0.7 | C3 |
| 3A5M | -0.9 ± 0.5 | -6.8 ± 0.7 | C2 |
| 3DKC | 12.0 ± 1.0 | -3.6 ± 1.3 | C3 |
| 3HY2 | 8.1 ± 0.5 | -3.1 ± 0.6 | C3 |
| 3IQ0 | 8.4 ± 0.4 | -5.3 ± 0.7 | C2 |
| 3KMW | 11.5 ± 0.4 | 1.8 ± 1.9 | C3 |
| 3M6G | 0.9 ± 0.7 | -9.4 ± 0.3 | C2 |
| 3QXC | 2.1 ± 0.8 | -10.6 ± 0.9 | C2 |
| 4B1W | -3.6 ± 1.6 | -8.0 ± 0.6 | C2 |
| 4EOM | 8.5 ± 1.0 | -6.4 ± 1.0 | C3 |
| 4FVR | 17.0 ± 1.4 | 2.6 ± 0.4 | C3 |
| 4USJ | 18.6 ± 0.8 | 7.5 ± 1.6 | C3 |

## S7. Allnér et al ion parameters

The reparameterisation of Allnér et al. (2012) addressed unphysically slow exchange rates of waters in the hydration shell of divalent cations, and of divalent cations bound to nucleic acids. We evaluated the effect of the modified $Mg^{2+}$ parameters on the C2/C3 free energy and the broader free energy landscape.

The large difference between Amber and CHARMM cation parameters (Allnér et al. 2012, Table 2) could account for some proportion of the large disagreement in the favoured coordination mode, presenting an informative test of force field sensitivity to the ion parameters. We note that the newly derived Mg2+ parameters are more similar to those of the CHARMM force field than to Amber, suggesting that the modification should show a larger effect on the Amber results.

The effect of the modified $Mg^{2+}$ parameters on the free energy as a function of Mg-αO distance is shown below. The free energy traces for each force field with modified $Mg^{2+}$ are coloured as shown in the legend, while the previous traces with unmodified $Mg^{2+}$ (main text, Figure 2 / Figure 7) are depicted alongside with desaturated colour. In accordance with the greater similarity of the new parameters with those of CHARMM, the effect on the free energy under both tested variants of the CHARMM force field is minimal, while the effect on the Amber force field is larger, with a calculated C3 to C3 free energy difference of +4.6 ± 0.2 kcal/mol as compared to 6.4 ± 0.1 kcal/mol for unmodified Amber. Again in accordance with expectations for a modification that more closely resembles unmodified CHARMM, the prediction is indeed shifted in the direction of the CHARMM prediction. Nonetheless, a large disparity between the Amber and CHARMM predictions persists.

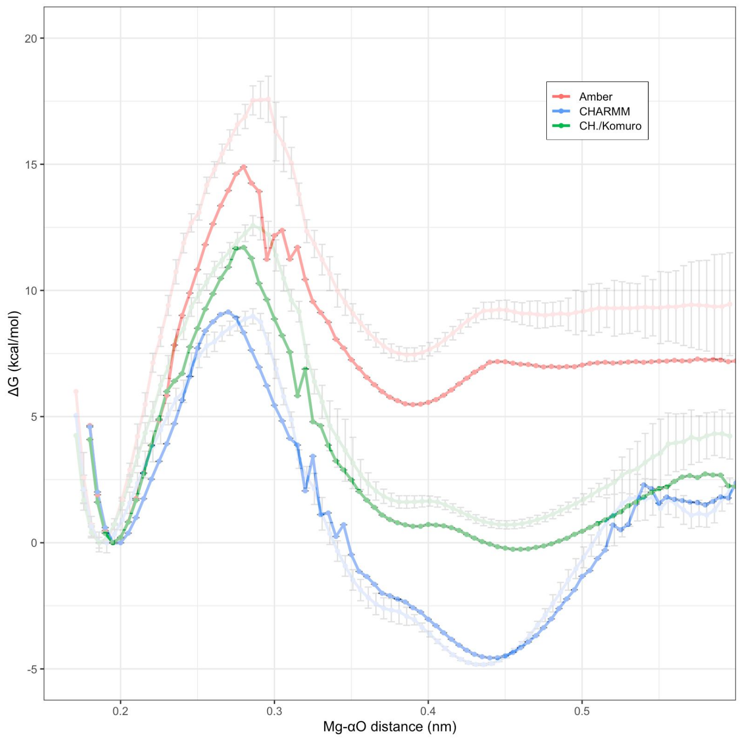

Finally, we compare the PCA visualisations of the free energy landscape (left to right: Amber, CHARMM and CHARMM with Komuro et al. bonded parameter modifications), directly equivalent to those in Figures 5a, 5b and 7b respectively. Here we also note only minor differences, with low free energy regions matching those observed for the unmodified force fields.

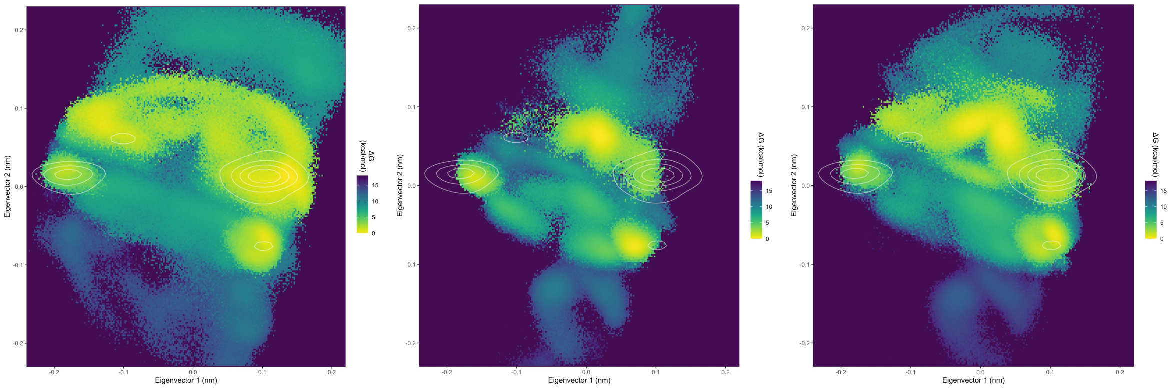


In summary, the Allner et al. $Mg^{2+}$ parameters bring the Amber C2/C3 balance marginally closer to experiment, and, as expected given the relative similarity of the newly derived parameters to those of CHARMM, to the CHARMM result. As we do not observe substantial changes to the broader free energy landscapes, our main findings for the tested force fields, as described under Conclusions in the main text, are equally applicable when using the modified $Mg^{2+}$ parameters of Allner et al..

## References


(1) Abraham, MJ; Murtola, T; Schulz, R; Páll, S; Smith, JC; Hess, B; Lindahl, E. GROMACS: High performance molecular simulations through multi-level parallelism from laptops to supercomputers. *SoftwareX* **2015**, *1-2*, 19-25.

(2) Darden, T; York, D; Pedersen, L. Particle mesh Ewald: An N · log(N) method for Ewald sums in large systems. J. Chem. Phys. **1993**, *98*, 10089-92.

(3) The PyMOL Molecular Graphics System, Version 2.4.0 Schrödinger, LLC.

(4) Liao, J.; Sun, S.; Chandler, D.; Oster, G. The conformational states of Mg.ATP in water. Eur. Biophys. J. **2004**, *33*, 29-37.
